# GRPAFusion: A Gradient Residual and Pyramid Attention-Based Multiscale Network for Multimodal Image Fusion

**DOI:** 10.3390/e25010169

**Published:** 2023-01-14

**Authors:** Jinxin Wang, Xiaoli Xi, Dongmei Li, Fang Li, Guanxin Zhang

**Affiliations:** 1 Optoelectronic System Laboratory, Institute of Semiconductors, Chinese Academy of Sciences, Beijing 100083, China; 2 College of Materials Science and Opto-Electronic Technology, University of Chinese Academy of Sciences, Beijing 100049, China

**Keywords:** image fusion, multimodal image, end-to-end model, gradient residual, pyramid attention

## Abstract

Multimodal image fusion aims to retain valid information from different modalities, remove redundant information to highlight critical targets, and maintain rich texture details in the fused image. However, current image fusion networks only use simple convolutional layers to extract features, ignoring global dependencies and channel contexts. This paper proposes GRPAFusion, a multimodal image fusion framework based on gradient residual and pyramid attention. The framework uses multiscale gradient residual blocks to extract multiscale structural features and multigranularity detail features from the source image. The depth features from different modalities were adaptively corrected for inter-channel responses using a pyramid split attention module to generate high-quality fused images. Experimental results on public datasets indicated that GRPAFusion outperforms the current fusion methods in subjective and objective evaluations.

## 1. Introduction

With the development of image sensor technology, it has become easy to obtain images at infrared wavelengths, which are invisible to the human eye, and at visible wavelengths. Infrared image sensors are noisy and have poor imaging quality; however, they can be used for imaging during the day as well as night. Visible image sensors have high image quality and clear textures but are susceptible to weather and environmental influences that prevent them from working around the clock. Multimodal image fusion is an effective means of image enhancement that can fuse useful information from different modal images into one image while removing redundant information. Fused images are easy for the human eye to see, and help to perform other downstream tasks such as object detection [1,2], object tracking [3], and object segmentation [4,5].

In recent years, researchers have proposed many traditional image fusion methods, including multiscale transform-based [6,7,8,9,10], sparse representation-based [11,12,13], and saliency map-based [14,15,16] methods. The multiscale transformation-based method transforms the original image according to the multiscale analysis method. The obtained transform coefficients are fused, and the corresponding inverse transform is performed to obtain the final fused image. The sparse representation-based method must construct a complete dictionary and then reconstruct the fused image according to the set fusion rules. The saliency map-based approach extracts a saliency map using saliency detection methods, and fuses the salient regions with other regions according to the fusion strategy. The fusion rules of these traditional methods are manually designed, and are computationally complex and time-consuming. In addition, these traditional methods do not consider the differences between different modalities, and use the same feature extraction method to extract the image features of different modalities, resulting in a large amount of redundant information in the fusion results, and poor fusion performance.

With the development of deep learning, researchers have proposed the use of convolutional neural networks (CNNs) for image fusion. The general steps of the deep learning-based method include feature extraction, fusion, and reconstruction. These methods include CNN-based [17,18], generative adversarial network (GAN)-based [19], and autoencoder (AE)-based [20] methods. Earlier, CNN-based methods used convolutional neural networks to only extract features, and they used traditional methods for image fusion, which is inefficient for fusion. Li et al. [21] proposed an end-to-end model for infrared and visible image fusion. The unsupervised network architecture avoided the use of fusion layers. However, this method used the same feature extraction layer to extract image features of different modes, resulting in an unbalanced feature representation of the reconstructed image to the original image. For example, the fused image is rich in texture information but lacks thermal-radiation information. Most autoencoder-based methods use manually designed fusion layers for feature fusion after feature extraction, and these manually designed fusion strategies limit fusion performance. The GAN-based approach makes it difficult to strike a balance between the generator and the discriminator, owing to the lack of ideal fusion images. To avoid this problem, Li et al. [22] proposed a dual discriminator network based on an attention mechanism. Instead of using the ideal fused image as the discriminator judgment criterion, this method used the saliency regions of different modal images as the discriminator criterion. This training method that only cared about local information made the fused images with artifacts and noise. Most current image fusion networks use only successive simple convolution operations for feature extraction and reconstruction, ignoring global dependencies and interchannel contextual information. SwinFusion [23], proposed by Ma et al. introduced the Swin transformer into image fusion to make full use of local and global information, and it achieved excellent fusion performance. However, the large number of parameters of transformer-based models brings a large computational overhead. Furthermore, multiscale feature extraction is a common means of obtaining global information. Frequently used methods include convolution with large convolution kernels or pooling operations with large strides. The convolution operation with large convolution kernels leads to an increase in computational effort and unavoidable block effects. The pooling operation causes a decrease in the image resolution, resulting in the loss of many vital details for image fusion tasks. Multi-task fusion frameworks have also made some progress in recent years. It is worth mentioning that SuperFusion [24], proposed by Tang et al., can implement image registration, image fusion, and segmentation tasks using only one framework.

This article proposes a multiscale image fusion framework called GRPAFusion, based on gradient residuals and pyramidal attention, to overcome the above problems. The framework is an end-to-end fusion network capable of extracting the features of different modalities based on the input image, thus performing adaptive image fusion. The fusion network is based on the encoder and decoder structures. The decoder uses a multiscale gradient residual (MGR) block to obtain more fine-grained multiscale information, with little computational effort, by increasing the receptive field. The pyramid split attention (PSA) module can acquire contextual information between the channels. The PSA module adaptively recalibrates the feature response between the channels, based on the dependencies between channels, to produce fused features that contain both infrared radiation and visible details. Adequate ablation and comparison experiments show that the proposed framework achieves the best fusion performance in subjective and objective evaluations.

**Figure 1:**
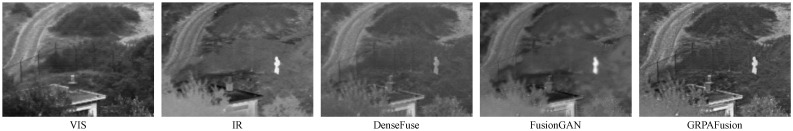
Example fusion results for FusionGAN, DenseFuse, and our GRPAFusion. GRPAFusion can retain both texture-detail information in the visible image and thermal-radiation information in the infrared image.

To visually demonstrate the excellent performance of the fusion framework proposed in this study, Figure 1 shows the comparison effect of the proposed method with the AE-based method DenseFuse and the GAN-based method FusionGAN fused images. The two images on the left are the visible and infrared images, and the middle image and the two images on the right are the fusion results of DenseFuse, FusionGAN, and GRPAFusion, respectively. The thermal-radiation information of the infrared image is not obvious in the fusion results of DenseFuse. The fused images of FusionGAN have considerable noise in the image due to an excessive focus on the infrared thermal information. In contrast, the fusion result of GRPAFusion proposed in this study includes thermal-radiation information in the infrared image, and preserves texture information in the visible image. The main contributions of this study are as follows:

1. We propose an end-to-end multimodal image fusion framework that can adaptively fuse information from different modalities without human intervention. The training process of the fusion network is constrained using adaptive pixel intensity loss and maximum gradient loss, such that the fused image can highlight the thermal-radiation information of the infrared image while retaining the texture details of the visible image effectively.
2. The fusion framework proposed in this study extracts the multiscale features of the source images while keeping the computational effort small and avoiding the use of downsampling operations. The detailed residuals of multiple granularities are used together in the encoder to represent the joint residual information, effectively preventing the problem of detailed feature loss and gradient disappearance. The pyramid split attention module is introduced in the feature fusion to make the fusion network pay more attention to the thermal-radiation information and texture details in the original image, whereas the fused image has higher contrast.
3. Adequate ablation and comparative experimental studies were conducted using the TNO dataset. The results of the ablation experiments show that the fusion framework and trained loss function proposed in this study are effective. The subjective and objective experimental results of the comparative experiments show that the multimodal image fusion framework proposed in this paper has superior fusion performance compared to current state-of-the-art image fusion methods.

The remainder of this paper is organized as follows. Section 2 describes the fusion framework in detail. Section 3 explains the experiments and discussions. Finally, Section 4 concludes the study.

## 2. Proposed Method

This section describes the multiscale image fusion framework based on gradient residual and pyramid attention in detail. The general structure of the fusion network is first described, then the loss functions we use and their functions are explained, and the evaluation method of the fused image quality is presented.

### 2.1. Network Architecture

**Figure 2:**
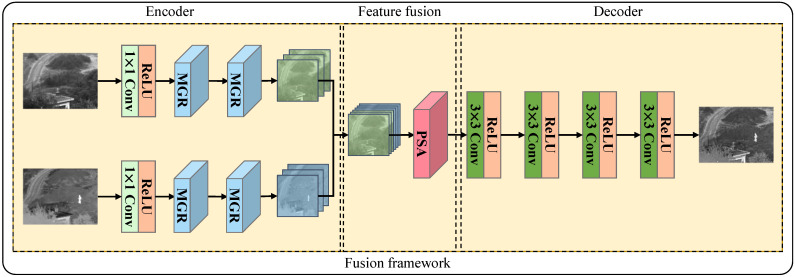
Overall structure of the fusion framework proposed in this article. The framework includes an encoder, feature fusion layer, and decoder.

The overall structure of the proposed fusion framework is shown in Figure 2, and it includes three parts: encoder, feature fusion, and decoder. In the image fusion process, the encoder first extracts the input image with multiscale depth features. It is fused with multiscale features using the PSA [25] module of the feature fusion layer, and finally, the final fused image is obtained after feature reconstruction via successive 3×3 convolutions.

**Figure 3:**
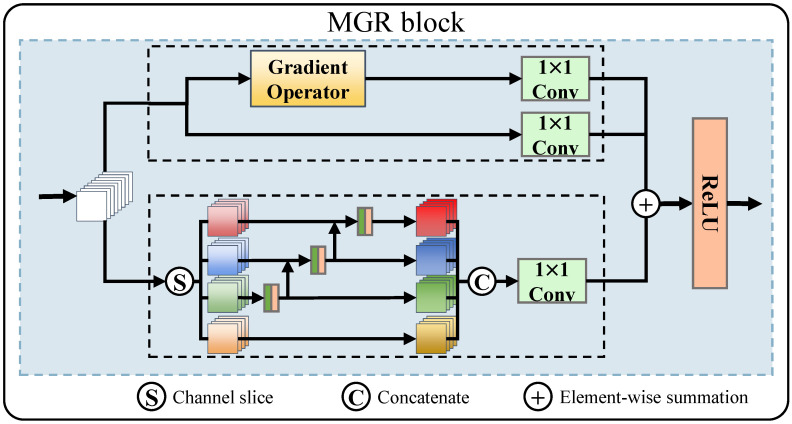
Structure of the MGR block. The MGR block is divided into two branches. The upper dashed box is the detailed branch used to extract multi-granularity detail features. The lower dashed box is the structure branch used to extract multiscale structural features.

**Encoder part:** We used 1×1 convolution and two MGR blocks to extract the image features. The structure of the MGR block is illustrated in Figure 3. The MGR block is divided into two branches. The upper part is called the detailed branch, and the lower is called the structure branch. The detailed branch is designed to extract multigranularity detail information, whereas the structure branch is designed to extract multiscale structure information. We used the original feature maps in the detailed branch as coarse-grained features. The fine-grained detail features in the image were extracted using the Sobel gradient operator. Then, after adjusting the feature channels using the 1×1 convolution method, the feature maps were added to the multiscale feature maps element-wise. Assuming that the initial feature map is *F*, the output of the detailed branch $F_{detial}$ is expressed as follows:

(1) $$F_{detial}=Conv_{1\times 1}\left(F\right)⊕Conv_{1\times 1}\left(\nabla F\right),$$

where $Conv_{1\times 1}\left(\cdot \right)$ represents the convolutional layer with a convolutional kernel size of 1, ⊕ stands for element-wise summation, and *∇* is the Sobel gradient operator. Inspired by Res2Net [26], in the structure branch, we acquired multiscale features by expanding the field of perception. The original feature map is first sliced into *s* feature groups according to scale *s*, denoted by $x_{i}$, where $i\in \left\{1,\;2,\;...,\;s\right\}$. In the method described in this article, *s* is 4. The feature map of the first group is directly output. The feature map of the second group is output after 3×3 convolution. The feature map of the third group is superimposed on the feature map of the second group, which is output after 3×3 convolution. After sequentially completing the operation of *s* groups of feature channels, a set of feature maps with multiple scales is obtained by cascading the *s* groups of feature maps between channels. Thus, the multiscale feature maps $F_{m}^{i}$ can be denoted as

(2) $$F_{m}^{i}=\left\{\begin{array}{ccc} x_{i} & \; & i=1;\\ Conv_{3\times 3}\left(x_{i}\right) & \; & i=2;\\ Conv_{3\times 3}\left(x_{i}⊕F_{m}^{i-1}\right) & \; & 2<i\leq s, \end{array}\right.$$

where $Conv_{3\times 3}\left(\cdot \right)$ represents the convolutional layer with a convolutional kernel size of 3. The output of the structure branch $F_{structure}$ is expressed as follows:

(3) $$F_{structure}=Conv_{1\times 1}\left(Concat\left(F_{m}^{i}\right)\right),\;i\in \left\{1,\;\;2,\;\;...,\;\;s\right\},$$

where Concat(·) denotes the concatenate operation on the channel dimension. Therefore, the output of the MGR block is expressed as follows:

(4) $$Out_{MGR}=\mathrm{ReLU}\left(F_{detial}⊕F_{structure}\right),$$

where ReLU(·) represents the linear rectification function. Two parallel channels are used in the encoder to extract the infrared and visible image features, respectively. The features of the two parallel channels are inter-channel cascaded to obtain the final output of the encoder.

**Figure 4:**
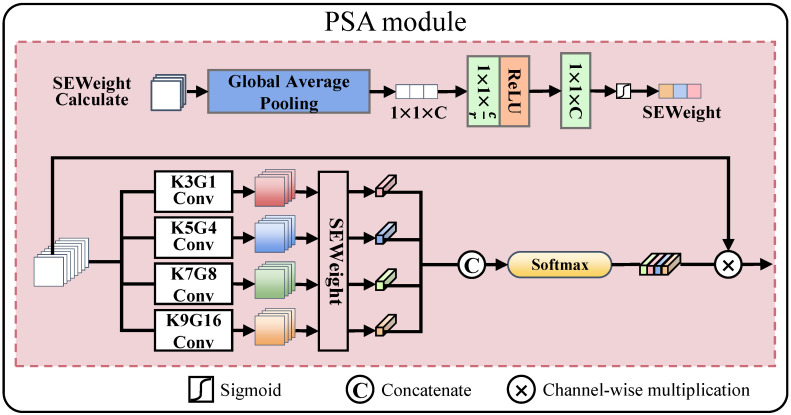
Structure of the PSA module. The PSA module uses convolution kernels of different sizes and group convolution to obtain multiscale attention maps, and adaptive fusion of different modal images is achieved by adjusting the response between feature fusion channels.

**Feature fusion part:** We use the PSA module in the feature fusion layer for multiscale feature fusion. The structure of the PSA module is shown in Figure 4. PSA uses four convolutional layers to divide the input fused feature map *F* into four feature subsets with a convolutional kernel size of K={3,5,7,9}. The number of channels is consistent for each feature subset. PSA uses group convolution to reduce the computational effort. The number of groups are G={1,4,8,16}. The pyramid feature map generation function is given by

(5) $$F_{pyramid}^{i}=Conv_{K_{i}}^{G_{i}}\left(F\right),\;\;i\in \left\{1,\;\;2,\;\;3,\;4\right\},$$

where $Conv_{K_{i}}^{G_{i}}\left(\cdot \right)$ represents the convolution operation with a convolution kernel size of $K_{i}$ and the number of groups $G_{i}$. The results are then passed through SEWeight [27], and the channel is cascaded to obtain a multiscale channel attention map $W_{att}$, which can be denoted as

(6) $$W_{att}=Concat\left(SEWeight\left(F_{pyramid}^{i}\right)\right),$$

where SEWeight(·) is used to obtain the attention weight from the input feature map. The interaction between global and local attention is implemented by SoftMax to obtain the final attention weights. Therefore, the output of the PSA module is expressed as follows:

(7) $$Out_{PSA}=F⊗\mathrm{Softmax}\left(W_{att}\right),$$

where ⊗ represents the channel-wise multiplication, and Softmax(·) is used to recalibrate the attention weight. The PSA module achieves an adaptive fusion of infrared and visible image features by adjusting the response between the feature fusion channels.

**Decoder part:** Decoder reconstructs the fused features to obtain the final fused image. Our decoder network consists of four convolutional layers. The convolutional kernels are all 3×3 in size, with a step size of 1. To avoid information loss, we obtain the final fused image via the successive convolutional adjustment of the feature channels without any up-sampling or down-sampling operations. This design allows our fusion network to accommodate image inputs of any size resolution, and feature maps and fused images can be output with the same resolution.

### 2.2. Loss Function

Infrared and visible image fusion is an enhancement method that aims to obtain a visible image that contains infrared thermal-radiation information and rich texture details. Therefore, this study proposes the use of content loss and detail loss as joint losses in the training phase to guide the optimization of network parameters. The total loss function is expressed as follows:

(8) $$L_{total}=L_{content}+L_{detial}.$$

Content loss is used to calculate the pixel intensity error between the fused image and the input image, and detail loss is used to calculate the edge texture difference between the fused image and the input image, which are, respectively, expressed as follows:

(9) $$\begin{array}{cc} L_{content} & =\frac{1}{HW}∥\left.I_{f}-\left(\alpha I_{ir}+\beta I_{vis}\right){∥}_{1}\right., \end{array}$$

(10) $$\begin{array}{cc} L_{detial} & =\frac{1}{HW}∥\left|\nabla I_{f}\right|\left.-\max\left(\left|\nabla I_{ir}\right|,\left|\nabla I_{vis}\right|\right){∥}_{1}\right., \end{array}$$

where $I_{f}$ indicates the fused image, $I_{ir}$ indicates the infrared image, *H* denotes the height of the image, *W* denotes the width of the image, α and β are hyperparameters, ∇ represents the Sobel gradient operation, and max(·) represents the maximum selection operation. ${∥\cdot ∥}_{1}$ represents the L1-norm, and the calculation is expressed as follows:

(11) $${∥I∥}_{1}=\sum_{i}^{H}\sum_{j}^{W}\left|I\left(i,j\right)\right|.$$

The maximum selection strategy is used for detail loss because the texture-detail information in the fused image must be the maximum set of texture details in the infrared and visible images. We intend that the proposed network adaptively selects the pixel intensities of the infrared image, and the visible image be displayed in the fusion results, so α and β are used to adjust the ratio of the infrared image to the visible image content. Therefore, appropriately selecting α and β can enable the fusion network to have a better fusion effect, and this part will be described in detail in the ablation experiment.

### 2.3. Evaluation of the Fusion Results

Subjective and objective methods are used for evaluating the fusion image quality. The subjective evaluation method is based on the visual perception of the human eye. The evaluation criterion is whether the fused image contains valid information from the original image and removes redundant information. The subjective evaluation method is highly random, and the evaluator cannot accurately distinguish minor differences between the fused and source images. Thus, in this study, we used a combination of subjective and objective evaluations to assess the quality of fused images. Eight objective evaluation indices were selected from different perspectives to evaluate the fused images objectively. The evaluation metrics based on fused images include information entropy (EN) [28], spatial frequency (SF) [28], and average gradient (AG) [29]. The evaluation metrics based on the fused and original images include fusion quality ($Q_{abf}$) [30], pixel feature mutual information (${\mathrm{FMI}}_{pixel}$), discrete cosine transform feature mutual information (${\mathrm{FMI}}_{dct}$), wavelet feature mutual information (${\mathrm{FMI}}_{w}$) [31], and multiscale structural similarity (MS-SSIM) [32]. These evaluation metrics can effectively reflect the ability of the fusion network to fuse visual information, structural information, and detailed texture information. Larger values of evaluation metrics indicate a better performance of the fusion method.

## 3. Experiments and Discussion

This section provides the experimental validation of the performance of the proposed fusion framework. First, the detailed parameters for training and testing are presented. Then, an ablation study is conducted, focusing on the effects of the choice of hyperparameters, and the network structure on the fusion performance. Finally, we evaluate the proposed fusion method qualitatively and quantitatively against current advanced fusion methods.

### 3.1. Experiments Setting

#### 3.1.1. Dataset

TNO [33] is an authoritative image fusion dataset, and we selected 21 representative pairs of scenes from the TNO dataset for ablation studies and comparison experiments. The data volume of the TNO dataset is small, to support the network’s training phase. Therefore, we selected LLVIP [34], which contains 14,588 pairs of infrared and visible images, to train the network.

#### 3.1.2. Train Details

In the training phase, we adjusted the resolution of the input image to 256×256, to speed up the training efficiency. The fusion network was trained on a PC containing an AMD R7-5800X 3.4 GHz CPU, 32 GB of RAM, and an NVIDIA GTX 3080Ti GPU. We used the deep learning framework Pytorch 1.8 and the Adam [35] optimizer to converge the loss function values to the minimum. The initial learning rate was 1e-4, the epoch was 4, and the batch size was 4.

#### 3.1.3. Test Details

We selected two traditional and four deep-learning-based fusion methods for comparison with our proposed method: ADF [6], MSVD [7], DLF [18], FusionGAN [19], DenseFuse [20], U2Fusion [36], SuperFusion [24], and SwinFusion [23]. Traditional methods were implemented using the MATLAB toolbox, and deep learning-based image fusion methods were implemented using publicly available source code. To ensure the fairness of the experiments, we tuned these methods to achieve the best performance according to the parameters recommended in the references.

### 3.2. Ablation Study

In the ablation study, we first performed a sensitivity-analysis experiment on the setting of hyperparameters, and determined the optimal parameter setting. Then, the ablation experiments of the network structure were performed to analyze the effects of the gradient residual and the PSA module on the network performance.

#### 3.2.1. Experimental Validation of Parameter Sensitivity Analysis

We use α and β as hyperparameters in the content loss function to adjust the ratio of infrared and visible image content. There are two ways to calculate α and β, based on global average pooling and global level map. The calculation based on the global level map method is as follows:

(12) $$\begin{array}{c} \alpha \left(x,y\right)=\frac{I_{ir}\left(x,y\right)}{I_{ir}\left(x,y\right)+I_{vis}\left(x,y\right)}, \end{array}$$

(13) $$\begin{array}{c} \beta \left(x,y\right)=\frac{I_{vis}\left(x,y\right)}{I_{ir}\left(x,y\right)+I_{vis}\left(x,y\right)}. \end{array}$$

**Figure 5:**
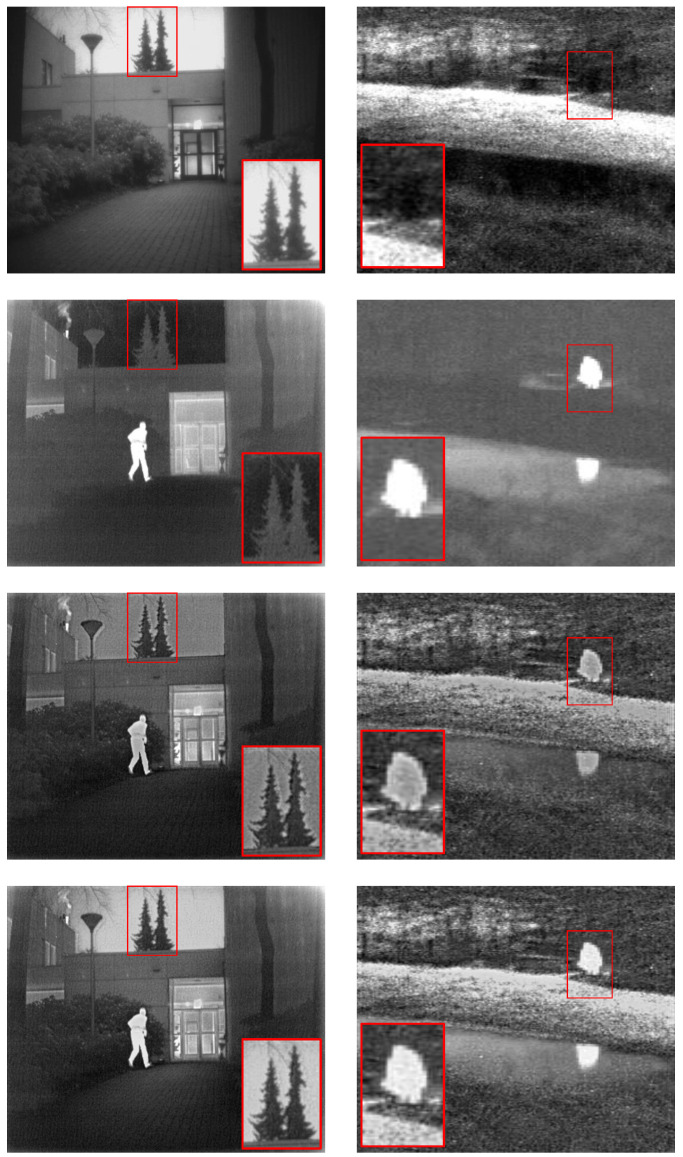
Representative results of the qualitative evaluation of the parameter sensitivity-analysis experiment. The scene on the left is *Kaptain_1123*, and that on the right is *Bench*. From top to bottom are the visible images, the infrared images, the fusion results based on the global average pooling method, and the fusion results based on the global level map method, respectively. The red boxes are used to mark key locations in the results.

The calculation based on the global average pooling method is as follows:

(14) $$\begin{array}{c} S_{u}=\frac{1}{HW}\sum_{x}^{H}\sum_{y}^{W}I_{u}\left(x,y\right), \end{array}$$

(15) $$\begin{array}{c} \alpha =\frac{S_{ir}}{S_{ir}+S_{vis}},\beta =\frac{S_{vis}}{S_{ir}+S_{vis}}, \end{array}$$

where *I* denotes the original image, and I(x,y) represents any pixel point in the image. With the network structure and other parameters being kept constant, we trained the network separately using the hyperparameters calculated via each of the above two methods. Figure 5 shows the representative results of the subjective experiments. The subjective experimental results show that the fusion results obtained using the network trained with the global level map-based approach to calculate hyperparameters are more prominent in the infrared thermal-radiation information and they avoid introducing additional artifacts. For example, in *Kaptain_1123*, the edges of the trees on the roof are sharper and free of artifacts, and the people on the *bench* are more prominent.

The average values of the objective evaluation metrics for 21 representative scenarios on the TNO dataset are listed in Table 1, with the best results in bold. The fusion results of the global-level map-based method outperformed those obtained using the global average pooling-based method for five of the eight objective evaluation metrics. In terms of the objective evaluation metrics, the fusion performances of the models obtained from the training of the global-level map-based approach were better. The hyperparameters chosen in the subsequent experiments in this study were calculated based on a global-level map.

#### 3.2.2. Experimental Validation of Network Architecture

**Figure 6:**
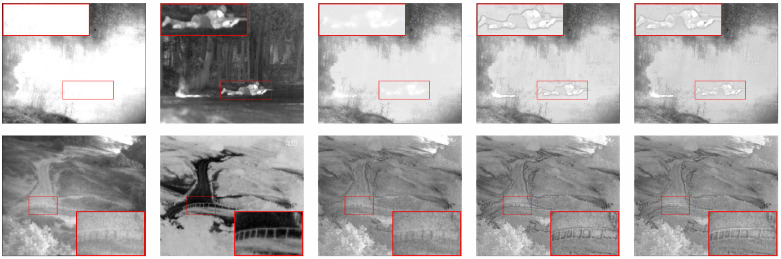
Representative results of the qualitative evaluation of the network-architecture validation experiment. The scene at the top is *soldier_behind_smoke*, and the scene at the bottom is *heather*. From left to right are visible images, infrared images, fusion results without gradient residual, fusion results without the PSA module, and our GRPAFusion, respectively. The red boxes are used to mark key locations in the results.

The fusion framework proposed in this study uses a gradient residual to provide more fine-grained, detailed information for the feature extraction part. It uses the PSA module as a feature fusion layer instead of the concatenate operation. The ablation study was conducted to analyze the impact of these two structures on the network performance. To verify the validity of the proposed network structure, we separately compared GRPAFusion with the model that excluded each of the two structures. The representative results of the subjective experiments are shown in Figure 6. In *soldier_behind_smoke*, fine-grained detailed features, such as the soldier’s body contour, are noticeably missing from the network fusion results without gradient residuals. In *heather*, the edges of the fence are clear and have less noise and artifacts in the fusion result of GRPAFusion. The fusion results using the PSA module as the fusion layer demonstrated higher contrast, and more realistic and natural images, because the PSA module can adaptively adjust the weight of infrared thermal-radiation and texture details during feature fusion.

The objective results of the network-structure validation experiments are presented in Table 2. Our proposed fusion network is optimal in all three structures in terms of metrics, except for EN and ${\mathrm{FMI}}_{w}$. The results of the subjective and objective experiments indicate that the structure of the fusion network proposed in this paper is effective. The results of the ablation study indicate that the gradient residual provides fine-grained detail features, and that the PSA module enables the fusion network to achieve adaptive image fusion.

### 3.3. Comparative Experiment

**Figure 7:**
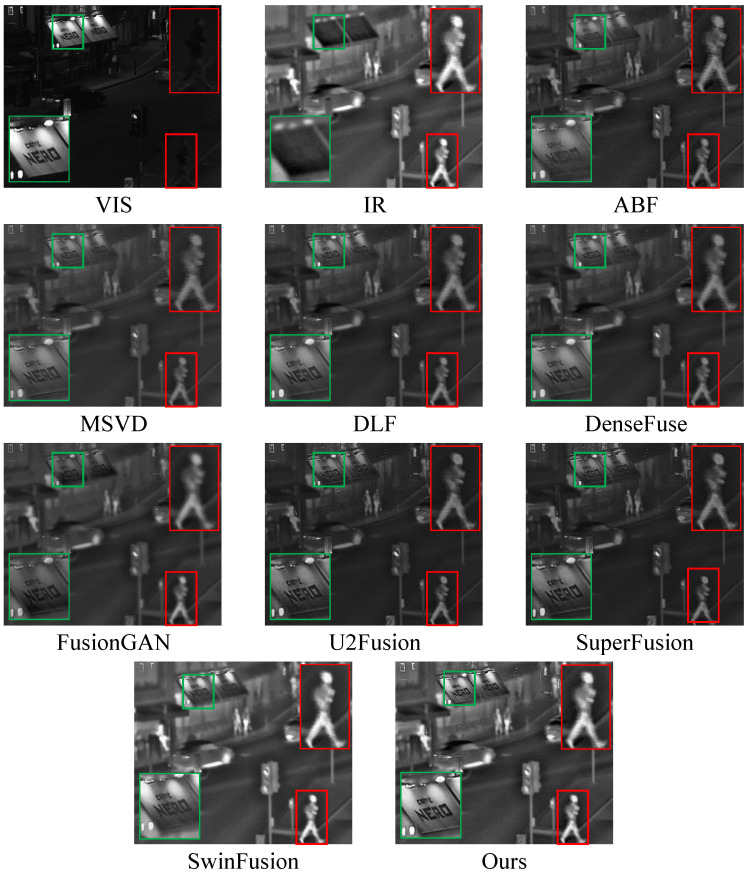
Subjective experimental results of comparison experiments on *Street* of the TNO dataset. Rectangular boxes were used to mark key locations in the results. The red boxes mark the infrared thermal information that requires attention, and the green boxes mark the texture details that require attention.

**Figure 8:**
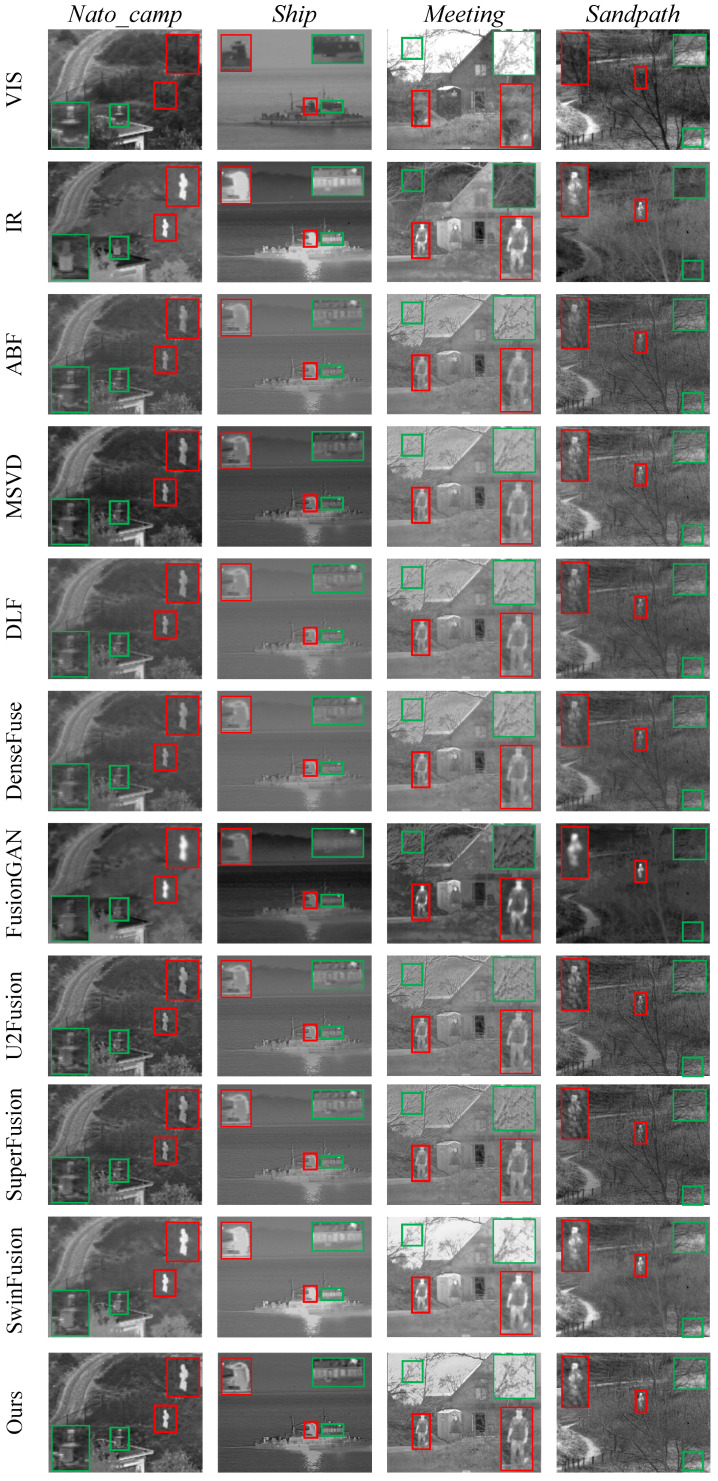
Subjective experimental results of comparison experiments on *Nato_camp*, *Ship*, *Meeting*, and *Sandpath* of the TNO dataset. Rectangular boxes are used to mark key locations in the results. The red boxes mark the infrared thermal information that requires attention, and the green boxes mark the texture details that require attention.

We conducted extensive comparative experiments on the TNO dataset to verify the excellent performance of the GRPAFusion. Figure 7 and Figure 8 show the representative results of the subjective experiments, from which we selected five representative scenes to demonstrate the excellent fusion effect of GRPAFusion. Rectangular boxes were used to mark key locations in the results. The red boxes mark the infrared thermal information that require attention, and the green boxes mark the texture details that require attention. In Figure 7, ABF, MSVD, DLF, DenseFuse, U2Fusion, and SuperFusion cannot clearly highlight the salient targets in infrared images, such as pedestrians on the road, with low luminance. FusionGAN can retain infrared thermal information; however, the fusion results contain considerable noise, resulting in blurred images. U2Fusion can preserve texture-detail information well; however, its ability to highlight infrared thermal information is weak. In the billboard of the *Street* scene, all comparison methods except U2Fusion and SwinFusion showed blurred text, whereas our method showed clear and sharp text with high contrast and a better fusion effect. In the *Meeting* scenario in Figure 8, artifacts appear to vary in degrees in the compared fusion methods, whereas our method avoids artifacts and provides a more realistic and natural fusion result. In the *Ship* scenario shown in Figure 8, the outline of the ship’s windows is significantly clearer in the fused image of GRPAFusion. The subjective experimental results indicate that GRPAFusion has better fusion performance, and the fused images can highlight the infrared saliency targets while retaining rich texture details. In the *Street* scene, GRPAFusion achieves the best subjective experimental result. However, the subjective experimental results in the *Nato_camp* and *Sandpath* scenes demonstrate that SwinFusion and GRPAFusion have similar fusion performances. To further compare the differences between the fusion performances of the comparison methods and GRPAFusion, we calculated the objective evaluation metrics for these methods.

**Figure 9:**
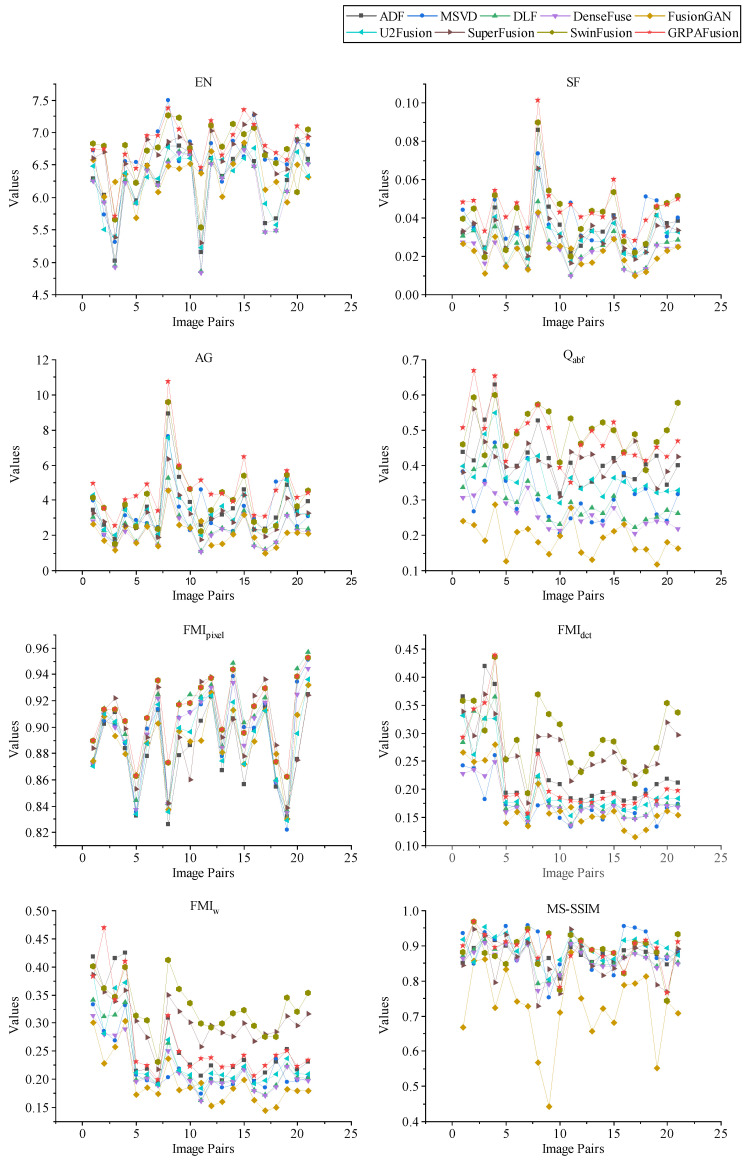
The results of eight objective evaluation metrics calculated on 21 pairs of representative images from the TNO dataset.

Figure 9 shows the objective evaluation results of the above fusion methods on the TNO dataset, where the solid red pentagrams represent the GRPAFusion calculations. As can be observed in the graph, the fusion results of the proposed method are significantly better than those of other methods on EN, SF, AG, and $Q_{abf}$. To evaluate its performance more intuitively, we calculated the average values of eight objective evaluation metrics for different fusion methods on the TNO dataset. The results are shown in Table 3, with the best results shown in bold and the second-best results underlined. The proposed method achieves the optimum in three objective metrics, EN, SF, and AG, indicating that GRPAFusion performs better in fusing effective information and preserving source image details. Suboptimal results were achieved for $Q_{abf}$ and ${\mathrm{FMI}}_{pixel}$, indicating that GRPAFusion can better preserve significant information in the source images. Although ${\mathrm{FMI}}_{dct}$, ${\mathrm{FMI}}_{w}$, and MS-SSIM did not achieve the optimum, GRPAFusion achieved optimality or suboptimality in most objective evaluation metrics. Therefore, combining the results of subjective and objective experiments, the fusion performance of GRPAFusion is better than those of other comparative methods.

## 4. Conclusions

In this study, we proposed an efficient end-to-end multimodal image fusion framework in which the trained network can adaptively fuse multiple modal images without human intervention. The fusion framework consists of three parts: encoder, feature fusion layer, and decoder. The source image is passed through the encoder section for multiscale and multi-grained feature extraction. The features of different modalities are fused and fed into the decoder for feature reconstruction to obtain the final fused image. In the training phase, we propose the use of content loss and detail loss to guide the convergence direction of the fusion network to make the final fused image have rich texture details and high contrast. The encoder uses an MGR block to extract multi-grained detail features and multiscale structural features, which can preserve the texture details of the source image. In addition, this study also introduced the PSA module instead of the simple channel cascade as the fusion layer, which adaptively fuses the features of different modes by readjusting the responses of different feature channels. Finally, the number of channels was adjusted using successive convolutions to obtain the fused image. The results of the ablation and comparison experiments on the TNO dataset indicated that GRPAFusion performs better than the state-of-the-art infrared and visible-image fusion methods. In the future, we will investigate better-performing modules to improve objective evaluation metrics, and we will further focus on how to use image fusion to drive advanced vision tasks.

## Figures and Tables

**Table 1:** Objective experimental results for different parameter settings on the TNO dataset; the best results are presented in bold.

Model	EN	SF	AG	$Q_{\mathrm{abf}}$	${\mathrm{FMI}}_{\mathrm{pixel}}$	${\mathrm{FMI}}_{\mathrm{dct}}$	${\mathrm{FMI}}_{w}$	MS-SSIM
Global Average Pool	6.6457	**0.0470**	4.6667	0.4778	0.9061	**0.2208**	0.2597	**0.9289**
Global Level Map	**6.8160**	0.0464	**4.6735**	**0.4818**	**0.9080**	0.2204	**0.2658**	0.8880

**Table 2:** Objective experimental results for different network architectures on the TNO dataset; the best results are presented in bold.

Model	EN	SF	AG	$Q_{\mathrm{abf}}$	${\mathrm{FMI}}_{\mathrm{pixel}}$	${\mathrm{FMI}}_{\mathrm{dct}}$	${\mathrm{FMI}}_{w}$	MS-SSIM
No-GradRes	**6.8677**	0.0453	4.3747	0.4669	0.9048	0.2171	**0.2744**	0.8649
No-PSA	6.8430	0.0460	4.5856	0.4756	0.9060	0.2153	0.2622	0.8730
Ours	6.8160	**0.0464**	**4.6735**	**0.4818**	**0.9080**	**0.2204**	0.2658	**0.8880**

**Table 3:** Objective evaluation results of comparison experiments on the TNO dataset, with the best results shown in bold and the second-best underlined.

Method	EN	SF	AG	$Q_{\mathrm{abf}}$	${\mathrm{FMI}}_{\mathrm{pixel}}$	${\mathrm{FMI}}_{\mathrm{dct}}$	${\mathrm{FMI}}_{w}$	MS-SSIM
ADF	6.2691	0.0345	3.5217	0.4127	0.8829	0.2275	0.2595	0.8760
MSVD	6.6088	0.0370	3.2704	0.3061	0.8960	0.1750	0.2191	**0.8933**
DLF	6.1855	0.0244	2.3587	0.2965	0.9033	0.1975	0.2251	0.8655
DenseFuse	6.1700	0.0219	2.2100	0.2627	0.8965	0.1767	0.2155	0.8603
FusionGAN	6.3600	0.0215	2.1173	0.1905	0.8889	0.1727	0.1957	0.7245
U2Fusion	6.2493	0.0313	3.3398	0.3655	0.8896	0.2054	0.2373	0.8931
SuperFusion	6.6180	0.0316	3.0343	0.4138	0.8961	0.2701	0.3031	0.8566
SwinFusion	6.7007	0.0408	3.9628	**0.4987**	**0.9102**	**0.2945**	**0.3267**	0.8844
Ours	**6.8160**	**0.0464**	**4.6735**	0.4818	0.9080	0.2204	0.2658	0.8880

## Data Availability

Not applicable.
